# Immunomodulatory Macrophages Enable E-MNC Therapy for Radiation-Induced Salivary Gland Hypofunction

**DOI:** 10.3390/cells12101417

**Published:** 2023-05-17

**Authors:** Ryo Honma, Takashi I, Makoto Seki, Mayumi Iwatake, Takunori Ogaeri, Kayo Hasegawa, Seigo Ohba, Simon D. Tran, Izumi Asahina, Yoshinori Sumita

**Affiliations:** 1 Department of Medical Research and Development for Oral Disease, Nagasaki University Graduate School of Biomedical Sciences, Nagasaki 852-8588, Japan; 2 Department of Regenerative Oral Surgery, Unit of Translational Medicine, Nagasaki University Graduate School of Biomedical Sciences, Nagasaki 852-8588, Japan; 3 CellAxia Inc., Tokyo 103-0012, Japan; 4 Department of Surgical Oncology, Nagasaki University Graduate School of Biomedical Sciences, Nagasaki 852-8588, Japan; 5 Laboratory of Craniofacial Tissue Engineering and Stem Cells, Faculty of Dental Medicine and Oral Health Sciences, McGill University, Montreal, QC H3A 1G1, Canada

**Keywords:** salivary gland, radiation-induced damage, cell therapy, macrophage, peripheral blood mononuclear cells

## Abstract

A newly developed therapy using effective-mononuclear cells (E-MNCs) is reportedly effective against radiation-damaged salivary glands (SGs) due to anti-inflammatory and revascularization effects. However, the cellular working mechanism of E-MNC therapy in SGs remains to be elucidated. In this study, E-MNCs were induced from peripheral blood mononuclear cells (PBMNCs) by culture for 5–7 days in medium supplemented with five specific recombinant proteins (5G-culture). We analyzed the anti-inflammatory characteristics of macrophage fraction of E-MNCs using a co-culture model with CD3/CD28-stimulated PBMNCs. To test therapeutic efficacy in vivo, either E-MNCs or E-MNCs depleted of CD11b-positive cells were transplanted intraglandularly into mice with radiation-damaged SGs. Following transplantation, SG function recovery and immunohistochemical analyses of harvested SGs were assessed to determine if CD11b-positive macrophages contributed to tissue regeneration. The results indicated that CD11b/CD206-positive (M2-like) macrophages were specifically induced in E-MNCs during 5G-culture, and Msr1- and galectin3-positive cells (immunomodulatory macrophages) were predominant. CD11b-positive fraction of E-MNCs significantly inhibited the expression of inflammation-related genes in CD3/CD28-stimulated PBMNCs. Transplanted E-MNCs exhibited a therapeutic effect on saliva secretion and reduced tissue fibrosis in radiation-damaged SGs, whereas E-MNCs depleted of CD11b-positive cells and radiated controls did not. Immunohistochemical analyses revealed HMGB1 phagocytosis and IGF1 secretion by CD11b/Msr1-positive macrophages from both transplanted E-MNCs and host M2-macrophages. Thus, the anti-inflammatory and tissue-regenerative effects observed in E-MNC therapy against radiation-damaged SGs can be partly explained by the immunomodulatory effect of M2-dominant macrophage fraction.

## 1. Introduction

Treatment of head and neck cancers with radiation therapy, including in combination with chemotherapy and/or surgery, causes a loss of fluid-producing acinar cells, leading to progressive tissue atrophy. This adverse effect irreversibly damages the function of the salivary glands (SGs) [1,2,3]. Affected patients typically suffer considerable morbid condition, as SG dysfunction results in a serious condition of xerostomia [1,2,3,4]. Xerostomia not only causes swallowing disorder but promotes dental caries, aggravates periodontal disease, and predisposes the patient to infections in oral and pharynx area [5]. Therefore, such conditions severely affect the patient’s quality of life [6]. Several recently developed approaches such as brachytherapy have shown merit in reducing radiogenic SG damage. However, a significant number of patients still undergo tissue atrophy and functional decline of SGs [7].

We developed novel therapeutic cells with enhanced vasogenic and anti-inflammatory functions from peripheral blood mononuclear cells (PBMNCs) via a new primary culture system (which we designated as “5G-culture”) using serum-free hematopoietic stem cell medium added five growth factors (Flt-3 ligand, Interleukin-6, stem cell factor, thrombopoietin, and vascular endothelial growth factor) [8,9]. The 5G-culture induces a heterogeneous cell fraction, which we named effective-mononuclear cells (E-MNCs), that include proliferated T helper 2 lymphocytes (Th2) and endothelial progenitor cells. Particularly, CD11b/CD206-positive M2-macrophages are specifically induced during the culture. We found that intraglandular transplantation of E-MNCs into radiation-induced atrophied SGs in a mouse model resulted in gradual recovery of saliva secretory function by 4 weeks post-irradiation (IR) [8]. Transplanted E-MNCs were detectable in the endothelium and glandular tissues up to 2 weeks after IR, but after 3 weeks, only in a few microvessels. Then, up to 12 weeks, E-MNC treatment affected the reduction of proinflammatory conditions in damaged SGs, and promotion of tissue regeneration, such as blood vessel formation and the proliferation of acinar and ductal stem/progenitor cells. Therefore, E-MNCs are excellent therapeutic cells supplied from a noninvasive source to regenerate SGs atrophied by radiotherapy. However, the detailed cellular mechanism of E-MNC treatment of atrophic SGs has not been elucidated.

To date, various experimental approaches of cell-based therapy employing gland epithelial stem/progenitor cells and/or mesenchymal stem cells (MSCs) have been examined for restoring the function of IR-damaged SGs [10,11,12,13,14,15,16]. Among these approaches, MSC therapy for atrophic SGs using intraglandular or intravenous injection of cells has been shown to slow the atrophic process by paracrine effects via induction of polarization of activated macrophages towards an anti-inflammatory phenotype (M2-type) at injury sites [13,14,15,16,17,18]. These macrophages contribute to revascularization and the following tissue regeneration [19,20,21]. Meanwhile, damage-associated molecular patterns (DAMPs) that flow out from dead cells into the extracellular environment trigger sterile inflammation after tissue injury, including injury caused by radiation therapy [22,23]. Such an inflammatory response promotes cell death, which leads to further release of DAMPs at injury sites [22,24,25,26]. However, enhancing DAMP clearance by infiltrating monocytes/macrophages that have shifted from a proinflammatory to anti-inflammatory phenotype via Mafb (a pivotal transcription factor for macrophage differentiation)-mediated induction of Msr1 (a scavenger receptor) expression resolves the sterile inflammation and prevents exacerbation of ischemic stroke pathology in the brain [27]. Thus, recent evidence suggests that therapeutic strategies that induce a shit from M1- to M2-macrophages effectively circumvent undesirable inflammation and promote tissue remodeling under certain conditions [28,29,30]. However, the accumulation of polarized M2-macrophages can promote fibrosis and the progression of chronic inflammatory diseases [31,32]. Therefore, achieving an appropriate balance between activated M1- and M2-macrophages at the injury site may be crucial for preventing the development of harmful conditions such as the overproduction of inflammatory (both pro- and anti-inflammatory) cytokines/chemokines [28,30]. As mentioned above, E-MNCs are enriched with a distinct population of (M2-macrophage-like) CD11b/CD206-positive cells, but CD206-negative (M1-macrophage–like) cells coexist in the CD11b expressing fraction of E-MNCs. The population of CD11b-positive cells, which includes both M1- and M2-macrophage–like cells in an appropriate activation state, may be essential for the therapeutic efficacy of E-MNC treatment.

As a prerequisite to clarifying the cellular mechanism of E-MNC treatment, this study focused on the CD11b-positive cell fraction among E-MNCs, which includes both M1- and specifically induced M2-macrophage-like cells, to investigate how this cell fraction contributes to the amelioration of damage in irradiated SGs. This study not only revealed the mechanism of E-MNC therapy but also might have uncovered a novel direction for cell therapies to treat atrophic diseases of the SGs.

## 2. Materials and Methods

### 2.1. Mice

Animal experiments in this study were carried out according to protocols approved by Nagasaki University Animal Care and Use Committee (approved number: 1605271307, 1610051411). As described in our previous study [8], female and male C57BL/6JJcl mice (eight weeks old) were used for recipients and donors, and C57BL/6-Tg (CAG-EGFP) male mice were applied as donors in some experiments to pursue the transplanted E-MNCs. 

### 2.2. Methods for 5G-Culture of PBMNCs 

#### 2.2.1. Mice Cells

**Figure 1:**
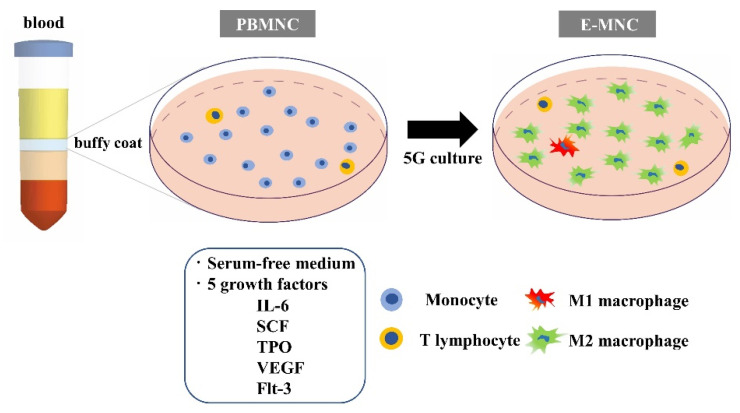
Schematic diagram of 5G-culture. Cultured PBMNCs were enriched in a population of CD11b-positive cells (M1- and specifically induced M2-macrophage-like cells).

To prepare E-MNCs, PBMNCs were cultured using the 5G-culture system, which was described in our previous work [8] (Figure 1). In brief, isolated PBMNCs were plated into wells of Primaria plates (BD Biosciences, San Jose, CA, USA) under specific conditions using Stem Line II medium (Sigma Aldrich, St. Louis, MO, USA) and 5 mouse recombinant proteins [8] (Supplemental Table S1). After 5 days, when CD11b/CD206-positive cells were maximally enriched, E-MNCs were collected for the following experiments. 

#### 2.2.2. Human Cells

Experiments were performed in compliance with the Helsinki Declaration. Blood samples were collected with permission from the Nagasaki University Ethics Committee (17082131). Written informed consent was obtained from donors. Healthy volunteer donors were four males aged 28 to 39 years. Human E-MNCs were generated using a specific 5G-culture method established by CellAxia Inc. (Tokyo, Japan), as described in our previous article [9]. Briefly, after density gradient centrifugation, separated PBMNCs were seeded to wells of Primaria plates and cultured in 5G-culture medium with 5 human recombinant proteins (Table 1). After 6–7 days, when CD11b/CD206-positive cells were maximally enriched, E-MNCs were harvested.

### 2.3. Evaluation of PBMNC and E-MNC Characteristics

For mouse cells, both PBMNCs and E-MNCs were evaluated by flow cytometry to identify macrophage subpopulations (M1 and M0: CD11b^+^/CD206^−^, and M2: CD11b^+^/CD206^+^) and T helper lymphocytes such as Th1, Th2, and Th17. In addition, this study assessed the immunomodulatory phenotypes of M2-macrophages using specific surface antigens (galectin3^+^/CCR2^−^/CD11b^+^ and Msr1^+^/CD11b^+^). For human cells, macrophage populations (M1 and M0: CD11b^+^/CD206^−^ and M2: CD11b^+^/CD206^+^) were also analyzed, along with propidium iodide (PI; the cell viability marker). Those antibodies are shown in Supplemental Table S2. Cell sample preparation and flow cytometric analysis were carried out as described in our previous study [8].

### 2.4. Immunocytochemistry of E-MNCs

Mouse PBMNCs were cultured in chamber slides (Millipore Millicell EZ slide; Merck, Darmstadt, Germany) at a density of 2.5 million cells/mL of 5G-culturemedium/well. After 5 days, E-MNCs were incubated with rat anti-mouse CD11b antibody (1:100; Abcam, Cambridge, MA, USA), rabbit anti-mouse CD206 antibody (1:100; Abcam), rabbit-anti mouse Msr1 antibody (1:200; Bioss, Woburn, MA, USA), or rabbit anti-mouse IGF1 antibody (1:200; Bioss). As secondary antibodies, Alexa Fluor 546–conjugated goat anti-rabbit antibody (1:200; Thermo Fisher Scientific Life Sciences, Waltham, MA, USA) for CD206, Msr1, and IGF1, and Alexa Fluor 647–conjugated goat anti-rat antibody (1:200; Cell Signaling Technology, Danvers, MA, USA) for CD11b were used. Mounting medium for fluorescence with 4′,6-diamidino-2′-phenylindole (DAPI; 1:1500; DOJINDO, Kumamoto, Japan) was employed for nuclear staining. 

### 2.5. Isolation of CD11b-Positive or -Negative Cells among E-MNCs

To determine the function of CD11b-positive macrophages among E-MNCs, positive cells (11b[+]) and negative cells (11b[−]) among E-MNCs were obtained using a magnetic cell separation system (autoMACS ProSeparator, ver.4.3.0; Miltenyi Biotec, Tokyo, Japan). Briefly, E-MNCs (mouse/human) were suspended in 90 µL (mouse)/80 µL (human) wash buffer (0.5% 2 mM EDTA-PBS buffer) per 1 × 10^7^ cells, and 10 µL (mouse)/20 µL (human) MicroBeads were added. After 30 min (mouse)/60 min (human) of incubation at 4 °C, the cells were washed in buffer and subjected to centrifugation for 10 min at 300× *g*. Finally, cells were suspended in 500 µL wash buffer, and clusters were removed using mini cell strainers II (Funakoshi, Tokyo, Japan) before proceeding to magnetic separation.

### 2.6. Analysis of Gene Expression in PBMNCs, E-MNCs, and Submandibular Glands

The mRNA expressions of samples were explored by real-time quantitative PCR as described in our previous study [8]. CD206, IL-1β, IL-6, IL-10, IGF1, TGF-β, TLR2, and TLR4 gene expressions were determined in mouse submandibular glands that were transplanted with mouse E-MNCs or 11b(−) cells (*n* = 3/each group at 10 days and 2 weeks of IR). The mRNA expressions of IL-1β, IFN-γ, and TNF-α in human PBMNCs stimulated with CD3/CD28 antibodies was also analyzed. In Supplemental Table S3, primer sets for mouse and human are shown.

### 2.7. Anti-Inflammatory Effects of Macrophages in E-MNCs under Co-Culture Conditions with CD3/CD28-Stimulated PBMNCs

Human PBMNCs were suspended in RPMI with 10% FBS and stimulated with CD3 and CD28 antibodies (Invitrogen, Waltham, MA, USA) coated on wells of a 24-well plate (1 × 10^6^ cells/600 µL/well) at 37 °C for 1 h (Supplemental Figure S1). Prior to cell seeding, CD3 (15 ng/mL) and CD28 (5 ng/mL) antibodies were diluted and then added at 250 µL/well to a 24-well plate and incubated overnight. To evaluate the anti-inflammatory effects of E-MNC, 11b(+) cell, and 11b(−) cell fractions on stimulated PBMNCs, the cells suspended in RPMI with 10% FBS were co-cultured in a Transwell upper chamber (Corning, Corning, NY, USA) for 1 h at 37 °C. Finally, total RNA of PBMNCs was extracted. 

### 2.8. Phagocytosis Assay

Phagocytosis assays using fluorescent beads were performed according to the manufacturer’s instructions (Cayman Chemical latex beads; Cayman Chemical, Ann Arbor, MI, USA). Human E-MNCs suspended in RPMI were seeded into the wells of a 24-well plate at a density of 1 × 10^5^ cells/500 µL/well. Then, E-MNCs were incubated with fluorescent beads for 1, 2, or 3 h, after which phagocytosis of latex bead–rabbit IgG-FITC complexes was measured by flow cytometry. Counterstaining of nuclei in E-MNCs of the 1 h group was carried out with mounting medium for fluorescence with DAPI after fixation. 

### 2.9. Irradiation and Time Course of Transplantation

Irradiation to mouse SGs was performed as previously described in our study [8]. Briefly, female mice were anesthetized and restrained in a specific container, and then given a single dose of 12 Gy gamma rays to the head and neck area. This dosage of IR was determined based on our preliminary study examining 10, 12, 15, and 18 Gy of gamma-ray irradiation, which was aimed at inducing more than 50% reduction of salivary secretion without declining health (decreasing the body weight). Doses of 15 and 18 Gy were too harsh to keep the health, while a dose of 10 Gy reduced salivary flow only by 50%. The 12 Gy was a dose to induce approximately 77% reduction of salivary flow without declining health up to 5 weeks of IR, and mice survived more than 6 months post-IR. 

E-MNCs or 11b(−) cells were directly transplanted into mouse submandibular glands at 7 days (2 × 10^5^ cells/gland) or 4 weeks (2 × 10^5^ and 1 × 10^6^ cells/gland) post-IR as preventive or disease-established models. The glands were harvested at 0 and 10 days and 2, 5, 9, and 13 weeks after IR. For transplantation, mice were randomly assigned to four groups in a blinded fashion: (1) control no-IR (Ctrl group) (*n* = 24); (2) IR and administration of 5 µL Iscove’s modified Dulbecco’s medium (IMDM; Sigma Aldrich) (IR group) (*n* = 24); (3) IR and E-MNC transplantation with 5 µL IMDM (E-MNC group) (*n* = 24); and (4) IR and transplantation of 11b(−) cells with 5 µL IMDM [11b(−) group] (*n* = 19).

### 2.10. Salivary Flow Rate after Irradiation

For evaluating the function of saliva secretion (salivary flow rate; SFR) of SGs, saliva was collected as described previously [8]. Saliva volume was measured gravimetrically (mg (collected saliva weight)/min (10 min)/body weight (converted per 100 g body weight)). SFR was determined at 0 (non-IR), 1, 2, 4, 5, 9, and 13 weeks post-IR. At each time point, the body weight was also measured.

### 2.11. Histological Analysis

After harvesting the submandibular glands, sections of tissue samples were prepared and observed by staining with hematoxylin and eosin (HE) and Masson’s trichrome, as described in our previous work [8]. Fibrosis was assessed under a microscope at ×200 magnification via analysis of three random fields in a section per 5 sections/3 specimens/group in the IR, E-MNC, and 11b(−) groups at 9 and 13 weeks of IR. 

Enhanced green fluorescent protein (EGFP)-labeled E-MNCs and 11b(−) cells obtained from CAG-EGFP mice were administered into irradiated submandibular glands at 1 week post-IR. Tissues were harvested at 10 days and 2 weeks post-IR (3 and 7 days post-transplantation), and then immunohistological staining was performed as described previously [8]. Rat anti-mouse F4/80 antibody (1:150 Bio-RAD Laboratories, Hercules, CA, USA), rabbit anti-mouse CD206 antibody (1:200 Abcam), rabbit anti-mouse IGF1 antibody (1:100; Bioss), rabbit anti-mouse high-mobility group box 1 (HMGB1) antibody (1:150; Abcam), rat-anti-mouse Msr1 antibody (1:200; Gene Tex, Alton Pkwy, CA, USA), rat-anti mouse CD117/c-Kit antibody (1:50; R&D Systems, Minneapolis, MN, USA), and rabbit anti-mouse Sca1/Ly6A/E antibody (1:50; Abcam) were applied as primary antibodies. Two examiners then independently determined the number of F4/80-, CD206-, IGF1-, Msr1-, and Sca1/c-Kit-positive cells around transplanted E-MNCs or 11b(−) cells at ×200 magnification via analysis of three fields in a section per 5 sections/3 specimens/group.

### 2.12. Enzyme-Linked Immunosorbent Assay (ELISA)

Epidermal growth factor (EGF) and hepatocyte growth factor (HGF) concentrations in saliva (*n* = 3/group in IR, E-MNC, and 11b(–) groups) were analyzed at 9 and 13 weeks post-IR using ELISA kits (EGF; ab234560, Abcam. HGF; EK1217, BOSTER, Pleasanton, CA, USA) according to the manufacturers’ protocols.

### 2.13. Microarray Analysis

Microarray analysis using DAVID Bioinformatics Database functional-annotation tools (http://david.abcc.ncifcrf.gov/, accessed on 1 September 2020), specifically for functional annotation clustering analysis, was carried out. The analysis was carried out to assess differences in characteristics between mouse PBMNCs and E-MNCs and between submandibular gland samples in the E-MNC group and IR group at 2 weeks post-IR.

### 2.14. Statistics

Experimental values are presented as the mean ± standard error. Differences between means were analyzed by one-way analysis of variance. To find significant differences within groups, Tukey’s multiple comparison *t*-test was performed. *p* values < 0.05 were considered statistically significant.

## 3. Results

### 3.1. Characteristics of Human E-MNCs

**Figure 2:**
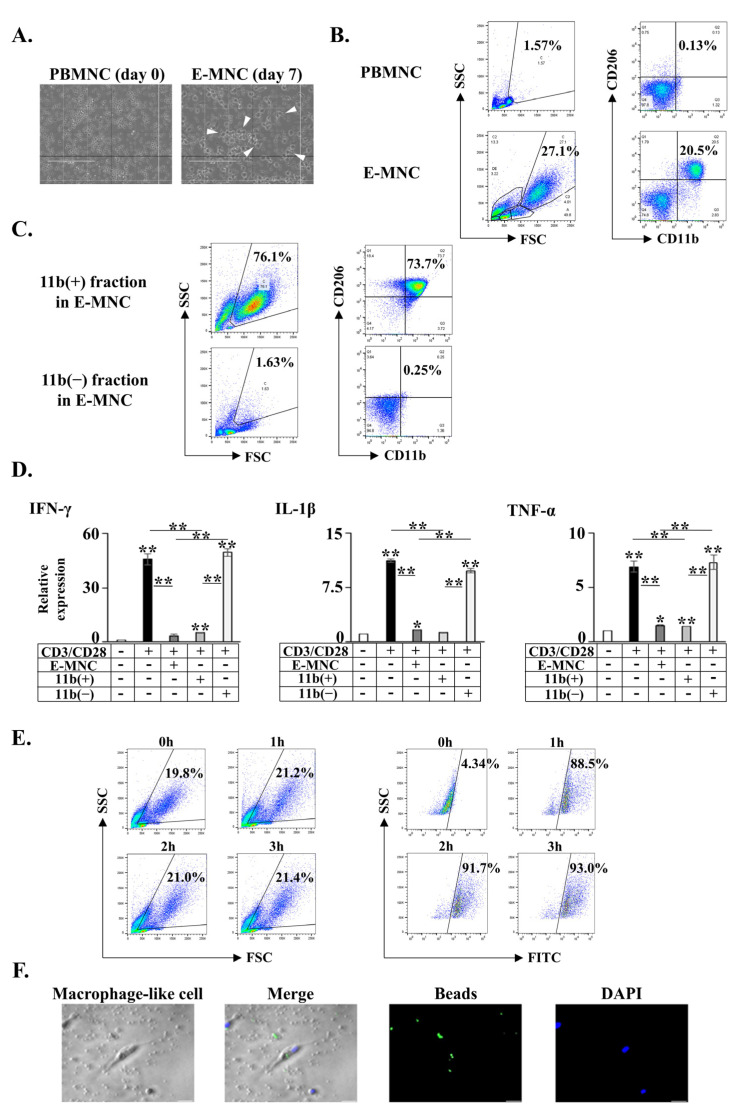
Characteristics of human E-MNCs. (**A**) Phase contrast images of MNCs. White arrows indicate round-shaped macrophage-like cells. Scale bar: 200 µm. (**B**) Analysis with flow cytometry. FSC/SSC gated cells, and M1; CD11b^+^/CD206^−^, M2; CD11b^+^/CD206^+^ macrophages among PBMNCs (day 0) and E-MNCs (day 7). (**C**) Flow cytometric analysis of FSC/SSC gated cells, and M1; CD11b^+^/CD206^−^, M2; CD11b^+^/CD206^+^ macrophages among CD11b-positive and -negative cell fractions after sorting CD11b-postive cells of E-MNCs with MACS. (**D**) mRNA expression in CD3/CD28-stimulated PBMNCs after co-culture with E-MNCs and CD11b-positive cells and CD11b-negative cells (* *p* < 0.05, ** *p* < 0.01). (**E**) Phagocytosis assay with fluorescent beads. Flow cytometric analysis of FITC-expressing cells at 0, 1, 2, and 3 h after addition of fluorescent beads (**right panel**) in FSC/SSC gated cells of E-MNCs (**left panel**). (**F**) Phagocytosis assay with fluorescent beads. Phase-contrast and fluorescence microscopic images of macrophage-like round- and spindle-shaped cells among E-MNCs at 1 h. Green: fluorescent beads; blue: DAPI. Scale bar: 20 µm.

After 7 days of culture, a proportion of adherent cells grew in size and their morphology altered to (macrophage-like) round and spindle forms (Figure 2A, Supplemental Figure S2A). Flow cytometry analysis showed a marked increase in FSC/SSC (cellular size/granularity) gated cells (from 1.96 ± 0.2% of PBMNCs to 24.75 ± 1.2% of E-MNCs) which comprise a larger cell population (such as macrophages) than other populations composed of lymphocytes or monocytes (Figure 2B). A fraction enriched in M2-macrophages (CD11b^+^/CD206^+^) emerged robustly among the E-MNCs (19.52 ± 0.62%), but this fraction was markedly smaller among PBMNCs (0.1 ± 0.02%) (Figure 2B). By contrast, the M1-macrophage (CD11b^+^/CD206^−^) ratio among E-MNCs exhibited minimal variation (2.83 ± 0.28%; no statistical differences were found between PBMNCs and E-MNCs). The M1/M2 ratio among E-MNCs was markedly reduced compared with the ratio among PBMNCs (M1/M2; from 10.9 to 0.14). 

When CD11b-positive cells (11b[+]) and -negative cells (11b[−]) were isolated using autoMACS to evaluate the anti-inflammatory function of CD11b-positive cells among E-MNCs, FSC/SCC gated cells accounted for 68.55 ± 3.14% of CD11b-positive cells, of which 56.63 ± 7.35% were M2-macrophages (CD11b^+^/CD206^+^). FSC/SCC gated cells accounted for 2.31 ± 0.31% of CD11b-negative cells, of which only 0.25 ± 0.02% were M2-macrophages (Figure 2C). Then, when cell fractions were applied to CD3/CD28-stimulated human PBMNCs, E-MNCs and CD11b-positive cells markedly suppressed IFN-γ, IL-1β, and TNF-α mRNA expressions, but CD11b-negative cells did not affect these expressions (Figure 2D). Assay using fluorescent beads was performed to investigate the phagocytic capacity of macrophages among E-MNCs. Although the ratio of FSC/SCC gated cells among E-MNCs did not change during the assay, fluorescent beads were engulfed by those cells over time (88.13 ± 0.56%, 91.73 ± 0.54%, and 92.33 ± 0.31% of gated cells after 1, 2, and 3 h, respectively) (Figure 2E). Microscopic observation revealed that round- or spindle-shaped macrophage-like cells had taken up the fluorescent beads (Figure 2F, Supplemental Figure S3A).

### 3.2. Phagocytic and Immunomodulatory Phenotypes of Mouse E-MNCs

**Figure 3:**
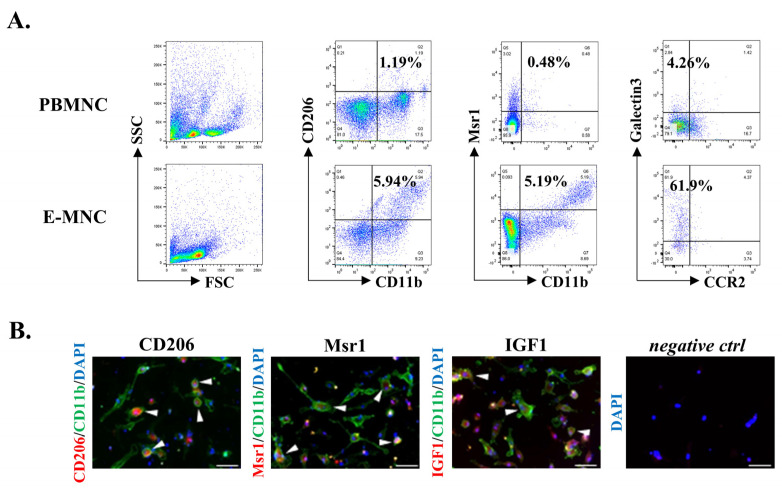
Characteristics of mouse E-MNCs. (**A**) Analysis of flow cytometry. M2; CD11b^+^/CD206^+^, M2c; CD11b^+^/Msr1^+^, and M2c; CCR2^−^/galectin3^+^ macrophages among PBMNCs and E-MNCs. (**B**) Analysis of the characters of CD11b-positive E-MNCs. Double immunofluorescence staining of CD11b cells (green) for CD206, Msr1, or IGF1 (red). Blue: DAPI; negative Ctrl (control), no primary antibody. Scale bar: 50 µm.

After 5 days, many adherent cultured cells grew in size and altered morphology to (macrophage-like) round or spindle forms [8]. Analysis by flow cytometry clearly showed the presence of a fraction (CD11b^+^/CD206^+^) putatively consisting of M2-macrophages among E-MNCs (from 0.96 ± 0.06% of PBMNCs to 5.17 ± 0.43% of E-MNCs) (Figure 3A). Furthermore, a population of Msr1 (a highly phagocytic M2-macrophage marker)-positive cells clearly appeared among E-MNCs (CD11b^+^/Msr1^+^; from 0.57 ± 0.05% of PBMNCs to 5.08 ± 0.73% of E-MNCs). In this regard, CD11b-positive cells among E-MNCs appeared to comprise an enriched cellular fraction of highly phagocytic macrophages that were induced to M2 polarization (CCR2^−^/Galectin3^+^; from 5.2 ± 0.27% of PBMNCs to 59.84 ± 2.71% of E-MNCs) (Figure 3A). For further characteristics of mouse E-MNCs, as shown in our previous study [8], M1-macrophages (CD11b^+^/CD206^−^) were largely diminished compared to those among PBMNCs, and Th2 cells (CCR4^+^/CCR6^−^) were markedly increased among T lymphocytes (CD3^+^/CD4^+^). Microarray analyses indicated that, overall, proinflammatory genes were downregulated in E-MNCs, whereas gene expressions related to angiogenesis, tissue regeneration, and M2-macrophages were upregulated when compared with PBMNCs (Supplemental Figure S4A). Immunocytostaining revealed that a number of adherent CD11b-positive macrophage-like cells expressed M2-phenotype markers (CD206 or Msr1) after 5G-culture, and production of IGF1, a cytokine that acts to resolve inflammation and mediates tissue regeneration, was observed in these cells (Figure 3B). 

### 3.3. Transplantation of E-MNCs or CD11b-Negative E-MNCs into a Prevention Model

**Figure 4:**
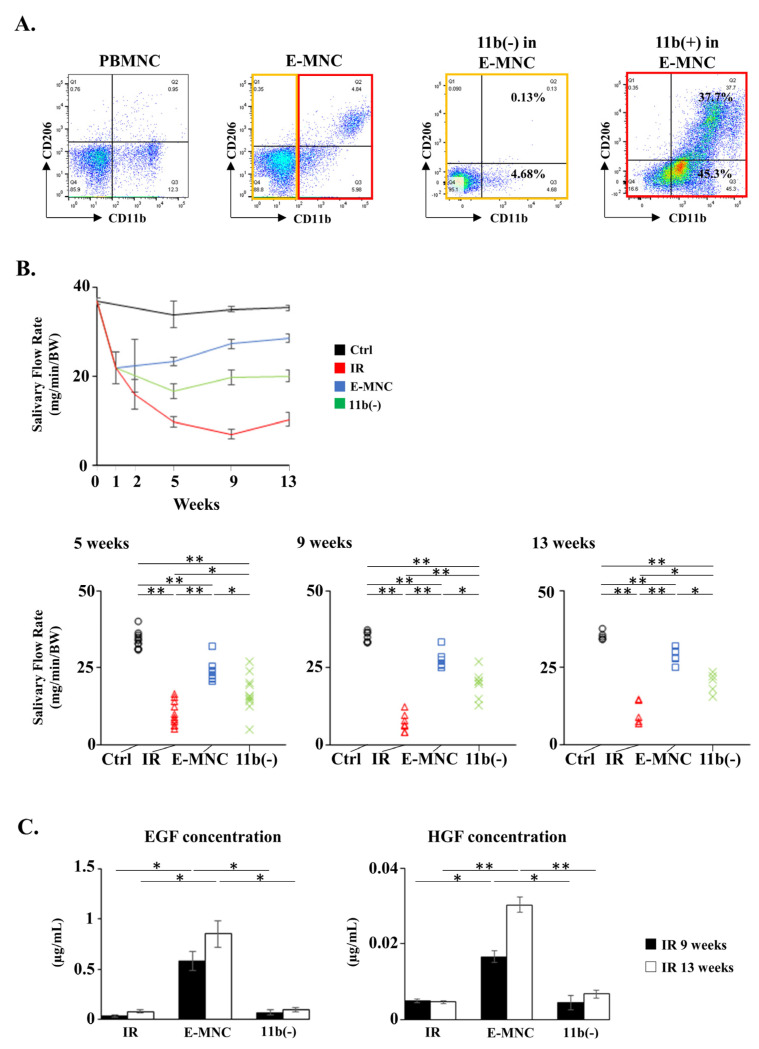
Transplantation of E-MNCs or CD11b-negative E-MNCs into a prevention model. (**A**) Analysis of flow cytometry. M1; CD11b^+^/CD206^−^) and M2; CD11b^+^/CD206^+^ macrophage fractions among PBMNCs, E-MNCs, CD11b-positive cells (red box area in E-MNCs), and CD11b-negative cells (yellow box area in E-MNCs), respectively. (**B**) Changes in saliva production (salivary flow rate; SFR) at 1, 2, 5, 9, and 13 weeks post-IR in each group. Lower graphs are scatter diagrams of SFR at specific time point (5, 9, and 13 weeks post-IR) for all groups (* *p* < 0.05, ** *p* < 0.01). (**C**) Concentrations of salivary EGF and HGF at 9 and 13 weeks post-IR (* *p* < 0.05, ** *p* < 0.01).

To examine the in vivo efficacy of CD11b-positive cells among E-MNCs, E-MNCs depleted of CD11b-positive cells (11b[−] cells among E-MNCs) were prepared, and then the macrophage fraction (CD11b^+^ cells), especially M2-macrophages (CD11b^+^/CD206^+^ cells), was confirmed to be nearly depleted from this fraction by flow cytometric analysis (Figure 4A). Saliva secretion decreased markedly for 1 week after IR and then continued to decline up to 9 weeks post-IR (IR group) (Figure 4B). In contrast, when E-MNCs transplanted at 1 week post-IR (E-MNC group), saliva output gradually recovered to ~80% of normal mouse (Ctrl group) levels up to 13 weeks. However, when depleted of CD11b-positive cells (11b[−] group), the efficacy of E-MNCs was reduced to a level ~50% lower than that of nondepleted E-MNCs at 5, 9, and 13 weeks post-IR (at 4, 8, and 12 weeks post-transplantation), respectively (Figure 4B). Meanwhile, the body weight of mice in the E-MNC group increased gently to the same level as that of mice in the Ctrl group, whereas mice in the CD11b(−) group gained a slight amount of body weight, similar to mice in the IR group (Supplemental Figure S5A). Finally, EGF and HGF concentrations in harvested saliva were significantly elevated in mice treated with E-MNCs at 9 and 13 weeks post-IR but not in mice treated with E-MNCs depleted of 11b-positive cells (Figure 4C).

### 3.4. Fibrosis in Submandibular Glands after Irradiation

**Figure 5:**
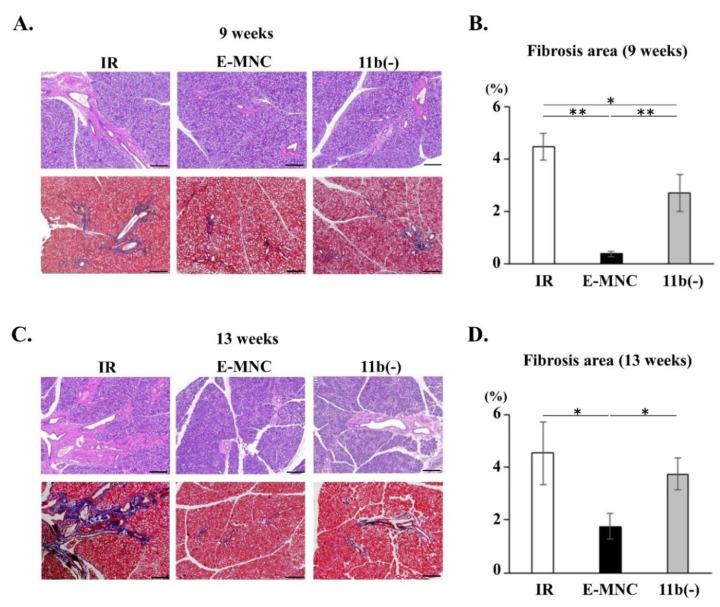
Fibrosis in submandibular glands after irradiation. (**A**) Histological analysis of submandibular glands at 9 weeks post-IR. Each panel shows representative images. Upper panels: HE staining; lower panels: Masson’s trichrome staining in each group. Fibrosis areas are stained blue. Scale bar: 200 µm. (**B**) Fibrosis area per field (%) in submandibular glands at 9 weeks (* *p* < 0.05, ** *p* < 0.01). (**C**) Histological analysis of submandibular glands at 13 weeks post-IR. Each panel shows representative images. Upper panels: HE staining; lower panels: Masson’s trichrome staining in each group in each group. Fibrosis areas are recognized as blue. Scale bar: 200 µm. (**D**) Percentage (%) of fibrosis area per field in submandibular glands at 13 weeks (* *p* < 0.05).

At 9 and 13 weeks, staining of HE and Masson’s trichrome clarified fibrosis in the submandibular glands (IR group) (Figure 5A–D). Meanwhile, fibrosis area in E-MNC-treated submandibular glands perceptible diminished compared to the IR group (0.08-fold at 9 weeks and 0.39-fold at 13 weeks post-IR) (Figure 5B,D). In contrast, the suppression of fibrosis was limited when CD11b-positive cells were depleted from E-MNCs (CD11b[−] group) (0.61-fold at 9 weeks and 0.83-fold at 13 weeks when compared to IR group) (Figure 5B,D).

### 3.5. Gene Expression in Submandibular Glands at 10 Days and 2 Weeks of IR

**Figure 6:**
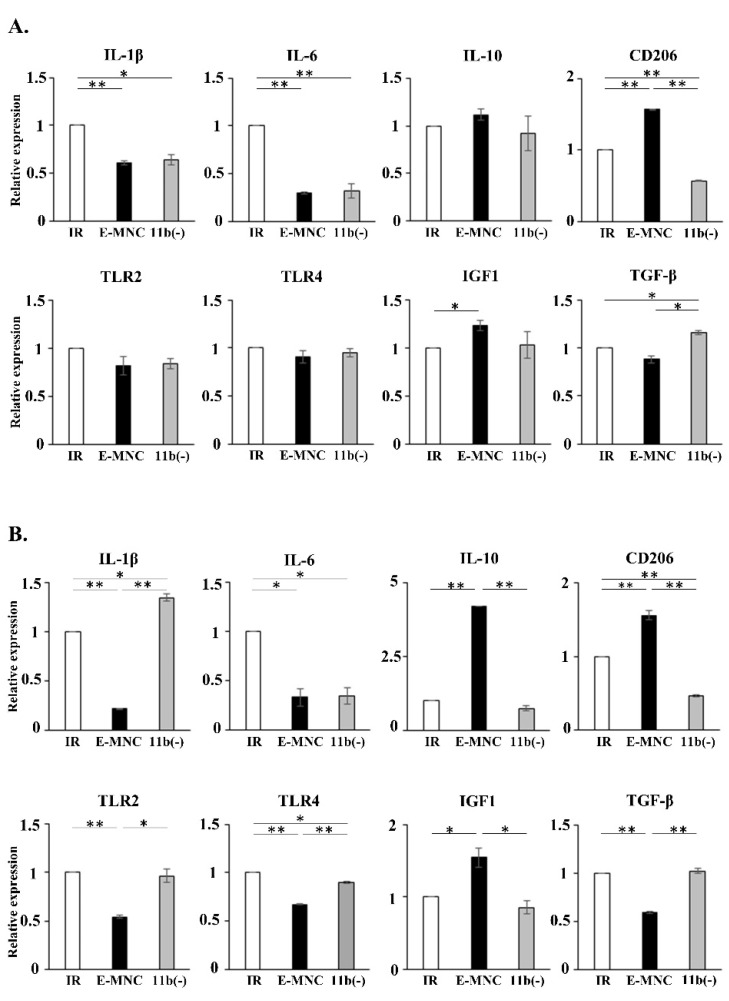
Gene expression in submandibular glands. (**A**,**B**) Expression of mRNAs of genes associated with proinflammation (IL-1β, IL-6, TLR2, TLR4), anti-inflammation (IL-10, CD206), tissue regeneration (IGF1), and fibrosis (TGFβ) in submandibular glands of nontreated (IR), E-MNC-treated, and CD11b(−)-treated mice at 10 days (**A**) and 2 weeks (**B**) of IR (* *p* < 0.05, ** *p* < 0.01).

At 2 weeks of IR (1 week post-transplantation), saliva output had already begun to recover, but only in the E-MNC group (Figure 4B). Therefore, as E-MNCs appeared to work effectively in damaged tissues from the initial stage of transplantation, SG specimens at 10 days and 2 weeks post-IR (3 days and 1 week post-transplantation) were analyzed. Gene expression analyses showed that E-MNCs and E-MNCs depleted of CD11b-positive cells (CD11b[−] cells) downregulated the proinflammatory gene expressions (IL-1β, IL-6) at 10 days, but no effect on Toll-like receptor (TLR) 2 and 4 mRNA expressions was observed at this stage (Figure 6A). However, while E-MNCs continued to inhibit IL-1β and IL-6 gene expressions at 2 weeks and also suppressed TLR2 and TLR4 mRNA expression, CD11b(−) cells did not inhibit mRNA expression of these genes, except for IL-6 (Figure 6B). IL-10 and CD206 mRNAs, which are associated with polarization of M2-macrophages, promoted their expressions in E-MNC-treated SGs at 10 days and 2 weeks of IR but were inhibited in SGs treated with CD11b(−) cells (Figure 6A,B). Consistent with these phenomena, E-MNC treatment increased IGF1 gene expression over time but downregulated the mRNA expression of TGF-β, which is associated with fibrotic activity, particularly after 2 weeks post-IR (Figure 6A,B). Microarray analyses of SGs at 2 weeks post-IR indicated that overall proinflammatory, matrix metalloprotease, and apoptosis-associated genes decreased their expressions, and upregulation of angiogenic and tissue regenerative genes were induced after E-MNC treatment compared with no cell transplantation (Supplemental Figure S6A). Among these genes, MMP9 mRNA expression was significantly suppressed by E-MNC treatment (0.15 ± 0.0076-fold compared with the Ctrl group) but not by CD11b depletion (Supplemental Figure S4B). Expression of mRNAs encoding NGF and Car3, which are involved in SG development/maintenance, was increased and maintained in E-MNC-treated submandibular glands versus IR or IR+CD11b(−) mice (NGF; 1.56 ± 0.16-fold over IR group, 1.79 ± 0.16-fold over CD11b[−] cells) (Car3; 9.56 ± 0.13-fold over IR group, 3.44 ± 0.13-fold over CD11b[−] cells) (Supplemental Figure S6B).

### 3.6. Immunohistological Observations in Submandibular Glands at 10 Days and 2 Weeks of IR

**Figure 7:**
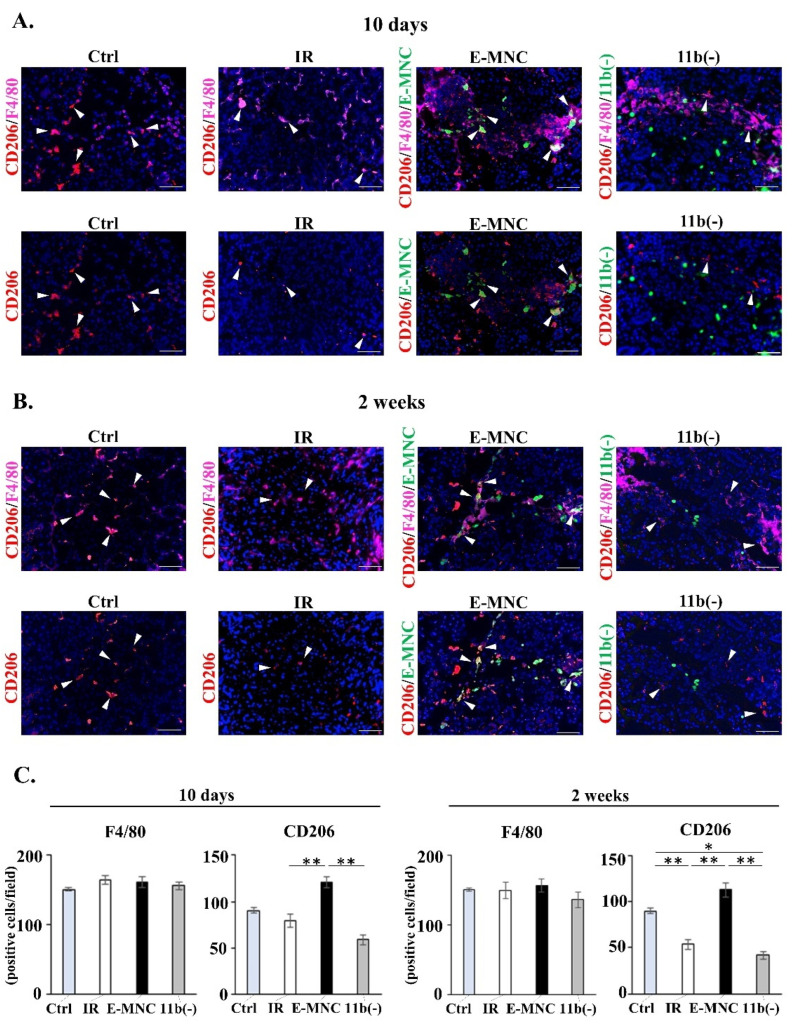
Detection of M2-macrophages in submandibular glands at initial stages after transplantation. (**A**,**B**) Double-stained images of F4/80 (pink) and CD206 (red) (**upper panel**) and single-stained images of CD206 (red) (**lower panel**) in tissues at 10 days (**A**) and 2 weeks (**B**) in each group. White arrow: F4/80^+^/CD206^+^ M2-macrophages. Green: transplanted E-MNCs or CD11b(−) cells. Blue: DAPI. Scale bar: 50 µm. (**C**) Graphs show the number of F4/80- and CD206-expressing cells in submandibular glands at 10 days and 2 weeks post-IR (* *p* < 0.05, ** *p* < 0.01).

Localization and quantification of specific subpopulations of macrophages in damaged tissue was visualized by immunohistochemistry. At 10 days post-IR, while there were no differences between groups in the number of F4/80 (a pan macrophage marker)-positive cells, E-MNC-treated mice had a higher number of CD206/F/4/80 (as M2-macrophages)-positive cells than mice in the other groups (Figure 7A,C). In particular, CD206-expressing host cells were seen at the periphery of EGFP-labeled E-MNCs containing CD11b-positive cells (Supplemental Figure S7A). Subsequently, at 2 weeks post-IR, E-MNC-treated mice still maintained a number of CD206-positive cells, whereas IR or IR+CD11b(−) mice largely exhibited a decrease in positive cells from 10 days post-IR (Figure 7B,C). 

**Figure 8:**
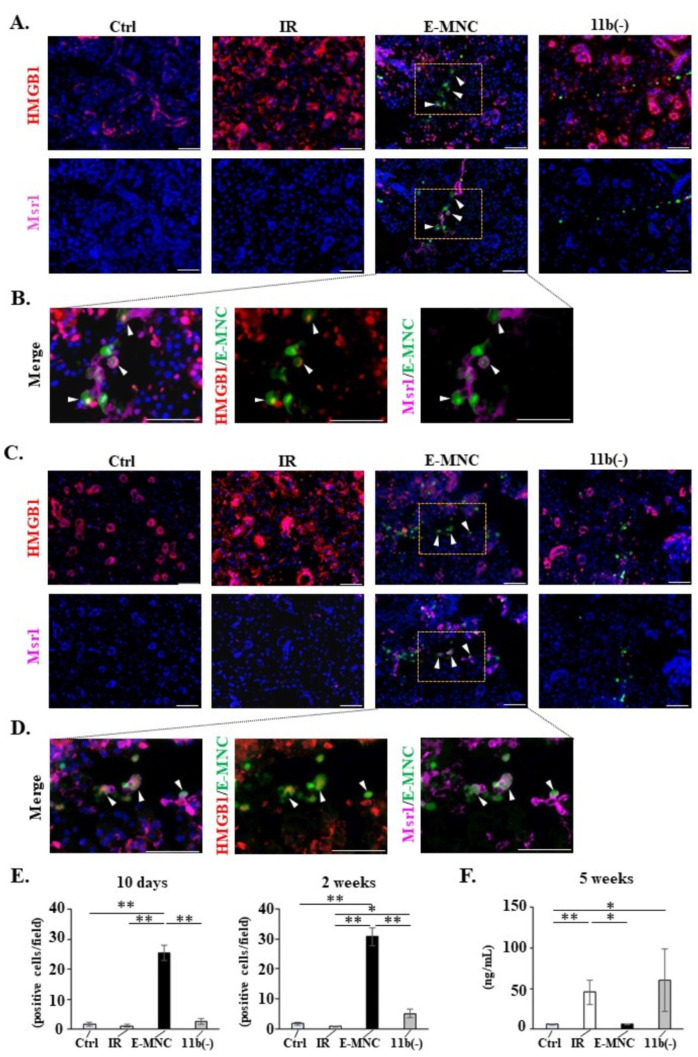
Detection of HMGB1 clearance in submandibular glands in the initial stages after transplantation. ((**A**,**C**) **upper panel**) Detection of released HMGB1 (red) in submandibular glands at 10 days (**A**) and 2 weeks (**C**) post-IR. Green: transplanted E-MNCs or CD11b(−) cells. Blue: DAPI. Scale bar: 50 µm. ((**A**,**C**) **lower panel**) Detection of Msr1-positive cells (M2c-macrophages) (pink) in submandibular glands at 10 days (**A**) and 2 weeks (**C**) post-IR. White arrow: Msr1-positive donor (EGFP^+^) cells. Green: transplanted E-MNCs or CD11b(−) cells. Blue: DAPI. Scale bar: 50 µm. (**B**,**D**) Merged images of HMGB1 (red) and Msr1 (pink) staining (yellow box areas in (**A**,**C**)) in E-MNC-injected SGs at 10 days (**B**) and 2 weeks (**D**) post-transplantation. White arrows: HMGB1^+^/Msr1^+^/EGFP^+^ E-MNCs. Blue: DAPI. Scale bar: 50 µm. (**E**) Graphs show the number of Msr1-positive cells in submandibular glands at 10 days or 2 weeks post-IR (* *p* < 0.05, ** *p* < 0.01). (**F**) Graph shows the concentration of HMGB1 in glands at 5 weeks (* *p* < 0.05, ** *p* < 0.01).

In investigating the presence of phagocytic M2-macrophages that function in clearing DAMPs from damaged tissues, increased numbers of Msr1 (a scavenger receptor of M2-macrophages)-positive cells were found at 10 days and 2 weeks of IR, but only in E-MNC-treated mice (Figure 8A–E). In addition, some Msr1-positive E-MNCs (EGFP-expressing cells) seemed to have internalized HMGB1 (as a representative DAMP) in E-MNC-treated tissues (Figure 8B,D). In contrast, abundant extracellular HMGB1 was detected in damaged tissues of IR or IR+CD11b(−) mice (Figure 8A,C). Indeed, at 5 weeks, HMGB1 concentration in E-MNC-treated glands was significantly reduced to levels similar to normal control glands compared with levels in IR or IR+CD11b(−) mice (0.31-fold and 0.13-fold, respectively) (Figure 8F).

**Figure 9:**
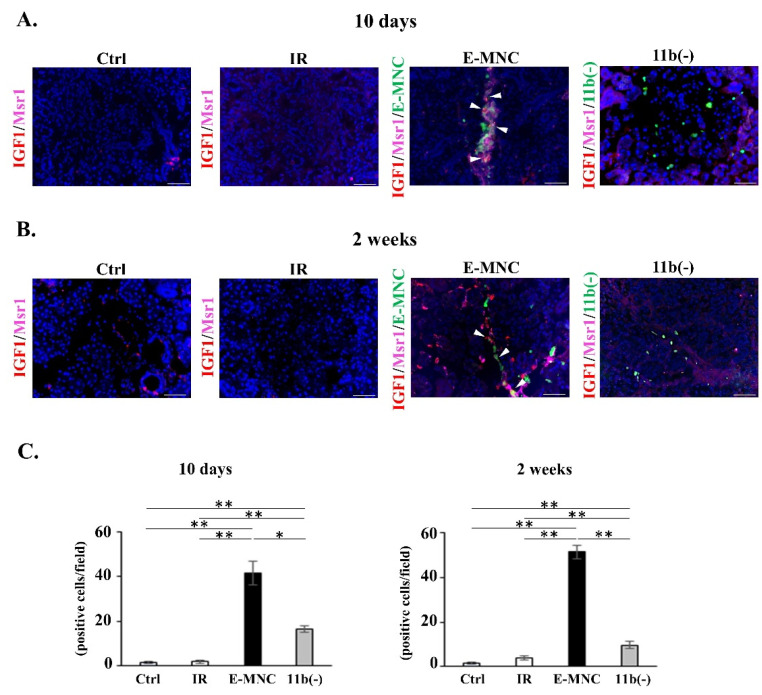
IGF1 production in phagocytic M2-macrophages in the initial stages after transplantation. (**A**,**B**) Detection of IGF1—(red) and Msr1 (pink)—expressing cells in gland tissues at 10 days (**A**) and 2 weeks (**B**) post-IR. Green: transplanted E-MNCs or CD11b(−) cells. White arrows: IGF1^+^/Msr1^+^/EGFP^+^ E-MNCs. Blue: DAPI. Scale bar: 50 µm. (**C**) Graphs show the number of IGF1-expressing cells in submandibular glands at 10 days or 2 weeks (* *p* < 0.05, ** *p* < 0.01).

**Figure 10:**
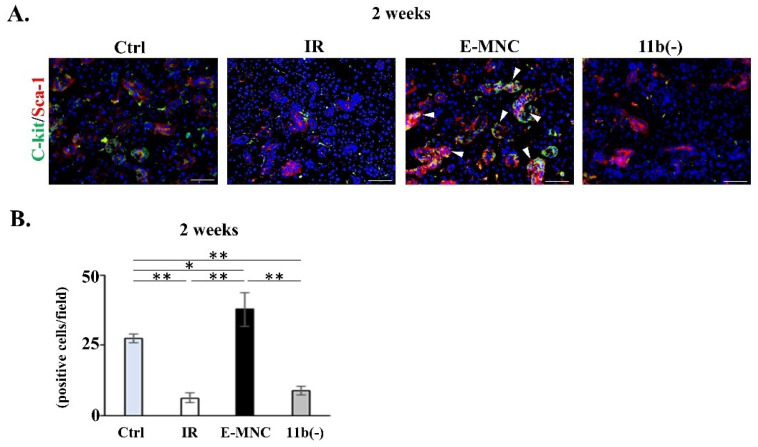
C-Kit/Sca-1 expressions in gland tissues at 2 weeks of IR. (**A**) c-Kit (green)/Sca-1 (red) expressions in each group. White arrows: c-Kit^+^/Sca-1^+^ (ductal stem/progenitor) cells. Blue: DAPI. Scale bar: 50 µm. (**B**) Number of c-Kit/Sca-1-expressing cells in each group (* *p* < 0.05, ** *p* < 0.01).

An assessment of tissue-regenerative activity revealed that some Msr1-positive E-MNCs (EGFP-expressing cells) expressed IGF1 in E-MNC-injected specimens at 10 days post-IR, and then scattered IGF1-expressing host cells were observed at the periphery of Msr1-positive E-MNCs after 2 weeks (Figure 9A,B). However, few IGF1- and Msr1-positive host cells were detected during this period in specimens transplanted with CD11b(−) cells. Quantitative analyses revealed that there were significantly IGF-1-expressing cells in both E-MNC and CD11b(−) groups, but the E-MNC-treated group was shown to be higher than the CD11b(−) group (approximately two- to fivefold) (Figure 9C). Consistent with these observations, c-Kit and Sca-1 double-expressing cells (as ductal stem/progenitor cells) were conspicuously recognized in duct cells of E-MNC specimens at 2 weeks post-IR (Figure 10A). Double-positive cells in the E-MNC-injected specimens were significantly higher in number than in other groups, while those cells decreased in specimens of IR and CD11b(−) groups compared to the Ctrl group (Figure 10B). 

### 3.7. Transplantation of E-MNCs or CD11b-Negative E-MNCs into a Mouse Model with Established Radiogenic Atrophic Salivary Glands

**Figure 11:**
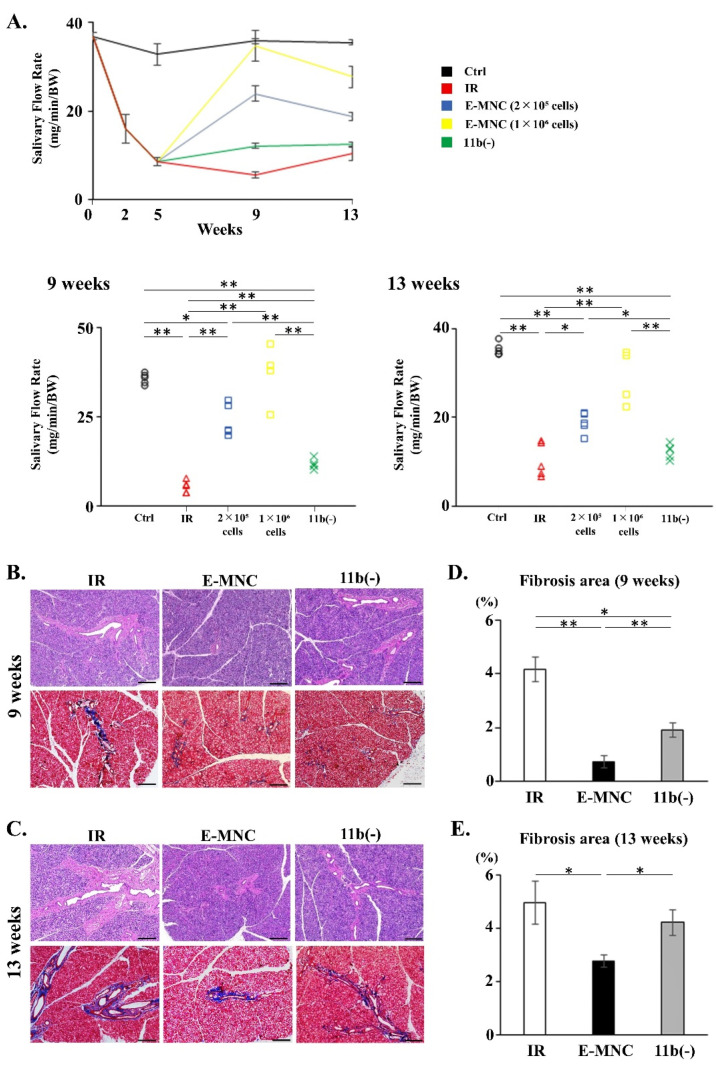
Transplantation of E-MNCs or CD11b-negative E-MNCs into an established model of radiation injury to salivary glands. (**A**) Changes in saliva production (SFR) at 2, 5, 9, and 13 weeks post-IR in each group. Lower figures, scatter diagram of SFR of each specimen in all groups at 9 and 13 weeks (* *p* < 0.05, ** *p* < 0.01). (**B**,**C**) Histological analysis of submandibular glands at 9 weeks (**B**) and 13 weeks (**C**) post-IR. Each panel shows representative images. Upper panels: HE staining; and lower panels: Masson’s trichrome staining in each group. Areas of fibrosis are stained blue. Scale bar: 200 µm. (**D**) Percentage of fibrosis area per field in gland tissues at 9 weeks after IR (* *p* < 0.05, ** *p* < 0.01). (**E**) Percentage of fibrosis area per field in gland tissues at 13 weeks of IR (* *p* < 0.05).

To further examine the efficacy of CD11b-positive cells among E-MNCs, E-MNCs (two doses of cell numbers for injection) and CD11b(−) cells were transplanted into damaged SGs at 5 weeks post-IR (4 weeks post-transplantation); this time point was chosen because hyposecretion of saliva induced by irradiation was established in mice (IR group) at this time point (Figure 11A). At 9 weeks post-IR, both the 2 × 10^5^ and 1 × 10^6^ E-MNC groups recovered saliva output to a level approximately 55% and 100% of that of normal mice (Ctrl group), respectively, whereas saliva output in nontransplanted mice (IR group) declined to a level approximately 17% of that of normal mice (Figure 11A). However, injection of 1 × 10^6^ E-MNCs maintained saliva output to a level 78.25% of that of normal mice at 13 weeks post-IR (Figure 11A). In contrast, when depleted of CD11b-positive cells (11b[−] group), the injection of 1 × 10^6^ E-MNCs reduced saliva output to a level ~25% of that of normal mice at 9 and 13 weeks post-IR. Overall, E-MNCs containing CD11b-positive cells were effective in restoring saliva production in damaged SGs. In terms of body weight, that of mice in the E-MNC group increased gradually but did not reach the level of Ctrl group mice, whereas mice in IR and CD11b(−) groups exhibited stable or slightly lower body weight from 5 to 13 weeks post-IR (Supplemental Figure S7B). 

Fibrosis in submandibular glands was observed in samples of IR group stained with HE and Masson’s trichrome at 9 and 13 weeks after IR (Figure 11B–E). However, the fibrosis area in submandibular glands injected with 1 × 10^6^ E-MNCs was clearly lower compared to that of nontreated glands (IR group) (0.18-fold at 9 weeks and 0.56-fold at 13 weeks post-IR) (Figure 11B,D). In contrast, the suppression of fibrosis was limited when CD11b-positive cells were depleted from E-MNCs (CD11b[−] group) (0.46-fold at 9 weeks and 0.85-fold at 13 weeks when compared to IR group) (Figure 11C,E). 

## 4. Discussion

This study showed that CD11b-positive cells play a vital role in determining the efficacy of E-MNC therapy against radiation-damaged SGs. The positive outcomes were as follows: (1) a CD11b-positive cell fraction composed of M1- and specifically induced M2-macrophages exhibited phagocytic and immunomodulatory activities in culture; (2) when depleted of this cell fraction, E-MNCs did not show sufficient efficacy in preventing fibrosis and stimulating recovery of saliva secretion in damaged glands; (3) CD11b-positive cells among E-MNCs and host M2-macrophages worked synergistically with regard to HMGB1 clearance and IGF1 secretion, and these actions appear to induce ductal stem/progenitor activation. These outcomes indicate that an M2-dominant macrophage fraction is an essential component of E-MNC-based cell therapy.

Regarding the phenotype and function of CD11b-positive cells among E-MNCs, CD206-positive M2-like macrophages were specifically induced in both human and mouse PBMNCs via 5G-culture. This culture method significantly reduced the M1/M2 ratio in a CD11b-positive cell fraction of PBMNCs (more than 50- and 10-fold lower in human and mouse E-MNCs). Particularly, in human E-MNCs, we found that CD11b-positive cells, which contained an abundance of M2-like macrophages (approximately 80% of CD11b-positive cells), were highly enriched (approximately 25% of E-MNCs). Indeed, similar to human E-MNCs, the CD11b-positive cells substantially inhibited the proinflammatory gene (IFN-γ, IL-1β, TNF-α) expressions in PBMNCs stimulated by T cell activation molecules in co-culture, whereas CD11b-negative cells among E-MNCs were unable to reduce the expression of these factors. In mouse E-MNCs, the abundance ratio of the M2-dominant CD11b-positive cell fraction was lower than that in human E-MNCs, but this cell fraction contained an abundance of Msr1- or galectin3-positive cells that could exhibit high phagocytosis ability associated with M2-macrophage polarization [29,33,34]. Meanwhile, Th2 cells were predominantly induced in T cell subsets after 5G-culture (from 0.1% in PBMNCs to 17% in CD3/CD4-positive E-MNCs), and such T cell differentiation over 5 days of culture appears to support the promotion of M2-macrophage polarization in CD11b-positive cells [30,35]. Indeed, expression of IL-10 and VEGF mRNA was upregulated in E-MNCs, whereas that of Th1-associated genes (IFN-γ and IL-1β) was downregulated. We then recognized that CD11b-positive cells produce IGF1, which is a primary factor in resolution of inflammation and polarization of M2-macrophages [36,37]. Moreover, M2-macrophages were recently shown to be a considerable source of IGF1 in immune metabolism and tissue regeneration [38]. These findings strongly suggest that CD11b-positive cells among E-MNCs exhibit high phagocytic and immunomodulatory activities in sterile inflamed tissues after radiation therapy. A certain polarization state of M2-macrophages in this CD11b-positive cell fraction, which in humans comprise approximately 80%, may govern the resolution of inflammation and stimulation of tissue regeneration [28,30].

With regard to the efficacy of E-MNC transplantation in treating radiation-damaged SGs, E-MNCs promoted recovery of saliva secretion and prevented the development of tissue fibrosis when transplanted not only in the early stage after irradiation but also in the late stage (after damage is established). E-MNCs depleted of CD11b-positive cells could not sufficiently rescue SG function. In particular, when transplanted in the late stage, the therapeutic effect was markedly reduced. As mentioned above, the abundance ratio of CD11b-positive cells was approximately 10% among mouse E-MNCs. Hence, the isolation and transplantation of only CD11b-positive cells has been technically challenging. However, once this cell fraction was depleted from E-MNCs, the efficacy of E-MNC transplantation was markedly impaired. Therefore, the CD11b-positive cell fraction appears to be essential for the therapeutic efficacy of E-MNC treatment. Indeed, E-MNCs functioned to maintain EGF and HGF concentrations in saliva at high levels for a long period and suppressed the development of tissue fibrosis, whereas E-MNCs depleted of CD11b-positive cells did not. EGF stimulates the proliferation of epithelial cells, and treating radiation-damaged SGs with MSC or other growth factors such as keratinocyte growth factor 1 (KGF1) leads to increased EGF secretion by acinar cells and higher resulting concentrations in saliva [13,39,40]. Likewise, HGF exerts anti-inflammatory or antifibrotic functions and was shown to exhibit the effects of protecting SG cells from radiation-induced cell death [41]. We have shown E-MNCs induce the proliferation of acinar and ductal cells during the regenerative process in radiation-damaged SGs [8]. Therefore, CD11b-positive macrophages among E-MNCs appear to affect the release of these growth factors from regenerated salivary epithelial cells.

To better understand the cellular mechanism of the in vivo efficacy of E-MNC treatment, this study explored how CD11b-positive macrophages work in damaged SGs. In particular, as we previously demonstrated that E-MNCs can be detected in damaged SG tissues up to 3 weeks after transplantation [8], we first analyzed the behavior of E-MNCs in the early stage of transplantation. We found that F4/80/CD206-positive cells (M2-macrophages) were significantly induced in E-MNC-treated SGs from 3 to 7 days post-transplantation. In contrast, these cells decreased over time in SGs treated with E-MNCs depleted of CD11b-positive cells. Interestingly, total number of F4/80-expressing cells (M1- and M2-macrophages) did not change in any group. Therefore, the abundance ratio of M2-macrophages increased robustly in damaged SGs in the early stage after E-MNC treatment. This phenomenon was likely caused by CD11b-positive cells among the E-MNCs, because many host M2-macrophages clustered around donor cells (CD11b-positive cells among E-MNCs)-derived CD206/EGFP-positive cells. As mentioned above, CD11b-positive cells among E-MNCs produce several cytokines, such as IL-10, VEGF, and IGF1. These proteins are associated with polarization of infiltrated monocytes/macrophages to the M2-phenotype at injury sites [30]. Meanwhile, we also found that the expression of HMGB1, a DAMP molecule, was increased in radiation-damaged SGs, and E-MNC treatment effectively decreased HMGB1 expression. HMGB1 was originally identified as a nuclear protein, but it is also known to play an essential role in mediating sterile inflammation when released to the extracellular environment from dead cells as a DAMP [29]. With regard to sterile inflammation, recent studies have reported that the radiation-induced innate immunity acts as an immune modulator [22,23,42]. Specifically, radiation leads to HMGB1 cytoplasmic translocation and extracellular release, and released HMGB1 in turn mediates the radiation-induced damage in normal tissues such as those in the lung via the TLR4 pathway [23]. Indeed, we preliminarily confirmed increased expression of TLR4 in ductal epithelial cells of mouse SGs after irradiation, accompanied by the extracellular release of HMGB1. Macrophages (both M1- and M2-macrophages) internalize extracellular DAMPs by binding their class A scavenger receptor (such as Msr1) [29]. However, HMGB1 is taken up efficiently by M2-macrophages rather than M1-macrophages. Such scavenger receptors on M2-macrophages are primarily functioned for HMGB1 clearance, whereas M1-macrophages secrete inflammatory cytokines in response to HMGB1 via the TLR4 pathway [29]. Indeed, internalization of HMGB1 by Msr1-positive cells among E-MNCs could be observed from the early stage of E-MNC transplantation. These cells showed the M2-phenotype and induced host Msr1-positive cells in damaged tissues, as indicated by the fact that not only Msr1-positive cells but also IGF1-expressing host cells increased around IGF1/Msr1-positive E-MNCs for up to 7 days post-transplantation. M2-macrophages are an important source of IGF1 production during tissue regeneration [34,38]. Previous studies have reported an aging-related decrease in IGF1 synthesis in SGs, resulting in lower levels of cell proliferation and tissue regeneration in oral tissues, and IGF1 injection prior to irradiation was shown to suppress apoptosis in acinar cells and prevent SG dysfunction in mice [43,44,45]. Therefore, these activities of the fraction of CD11b-positive cells among E-MNCs appear to induce the proliferation of c-Kit/Sca-1 expressing ductal progenitor cells. Additionally, the upregulated expression of mRNAs associated with SG tissue recovery, such as NGF and Car3, was recognized 7 days post-transplantation. Promotion of SG stem/progenitor cell activation from the early phase of sterile inflammation likely facilitates tissue regeneration without inducing the development of fibrosis.

## 5. Conclusions

**Figure 12:**
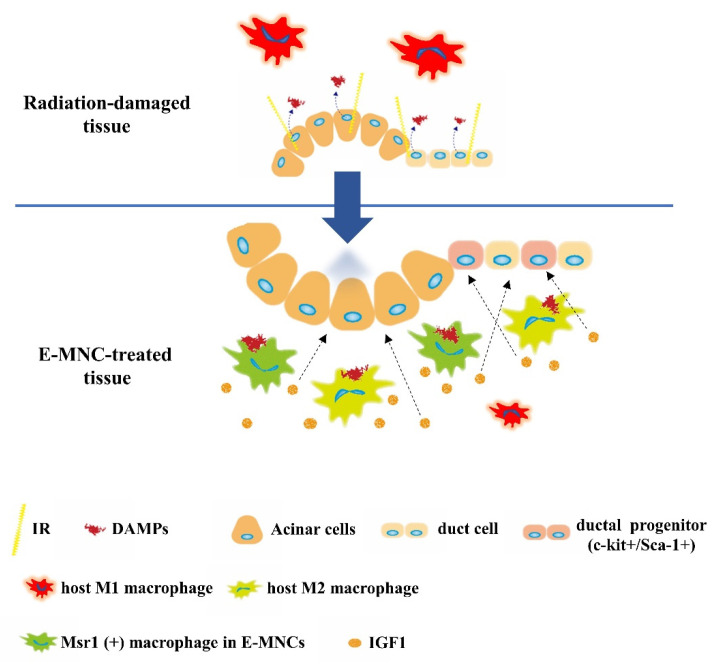
Schematic diagram of the suggested mechanism of E-MNC treatment. CD11b-positive cells among E-MNCs might contribute to convert the condition of damaged tissue from a proinflammation to an anti-inflammation and promote tissue regeneration by mediating DAMP clearance and IGF1 production in cooperation with host macrophages.

This study demonstrated that CD11b-positive cells, composed of M1- and specifically induced M2-macrophages, among E-MNCs contribute to suppress sterile inflammation and promote tissue regeneration by eliminating extracellular HMGB1 and inducing IGF1 production in radiation-damaged SGs (Figure 12). Overall, the mechanism underlying the effectiveness of E-MNC treatment for radiation damage in SGs can be explained in part by these findings. E-MNCs are a readily available and low-invasively obtained source of cells which can be produced within a week; thus, this therapy for treating radiogenic xerostomia can be easily performed in the clinic. However, the cellular mechanism should be investigated in greater detail for future clinical applications. This study was unable to clarify the detailed function and appropriate abundance of both M1- and M2-macrophages in the CD11b-positive cell fraction of E-MNCs, and, yet, CD11b-positive cells among human E-MNCs are composed mostly of M2-macrophages. Therefore, the predominant state of polarization toward M2-phenotype in the macrophage fraction must be at least essential for the function of E-MNC therapy. We are currently carrying out additional experiments to determine how significantly resolving sterile inflammation caused by DAMPs can serve as a therapeutic target for radiation-damaged SGs. In parallel, we are also investigating the intercellular interactions to initiate ductal stem/progenitor cell activation and acinar cell proliferation, required for tissue regeneration, via IGF1 production by donor and host M2-macrophages during the subsidence of sterile inflammation in damaged SGs. Another limitation of the present study is that a single-dose irradiation, which does not correlate with actual regimens of radiation therapy, was employed for revealing the exact mechanism of atrophied gland reconstruction by E-MNC treatment. Moreover, in this study, we employed a single transplantation using a certain dose to submandibular glands, which should not be enough to ensure actual treatment effects such as long-term efficacy. Therefore, future analytical experiments should be performed, employing proper models of clinical therapy using fractionated-dose irradiation with or without chemotherapy. Then, optimal therapeutic conditions to lead the clinical effectiveness, such as the frequency or dose of E-MNC transplantation, should be developed. This study found that an M2-dominant CD11b-positive macrophage fraction plays an essential role in E-MNC therapy. Our results suggest that host Msr1-positive macrophages are a potential target for newly developed therapeutics to facilitate atrophied SG regeneration.

## Figures and Tables

**Table 1:** Recombinant proteins of human 5G-culture medium.

Recombinant Proteins	Company, Catalog No.	Concentration
human SCF	Peprotech, 300-07-10UG	100 ng/mL
human Flt-3 ligand	Peprotech, 300-19-10UG	100 ng/mL
human TPO	Peprotech, AF-300-18	20 ng/mL
human VEGF	Peprotech, 100-20-100UG	50 ng/mL
human IL-6	Peprotech, AF-200-06	20 ng/mL

## Data Availability

The datasets are available upon reasonable request to the corresponding author.

## Supplementary Materials

The following supporting information can be downloaded at: https://www.mdpi.com/article/10.3390/cells12101417/s1, Table S1: Recombinant proteins of mouse 5G culture medium; Table S2-1: Mouse antibodies for flow cytometric analysis; Table S2-2: Human antibodies for flow cytometric analysis; Table S3-1: Mouse primers; Table S3-2; Human primers; Figure S1: Schematic diagram describing the experimental design for co-culture of E-MNCs and T cell-activated PBMNCs; Figure S2: Characteristics of human E-MNCs; Figure S3: Characteristics of human E-MNCs; Figure S4: Microarray analysis of PBMNCs and E-MNCs; Figure S5: Changes in body weight in the prevention mouse model after transplantation; Figure S6: Microarray and qPCR analyses of transplanted specimens at 2 weeks post-IR; Figure S7: Immunohistological observations at 10 days post-IR and changes in body weight after transplantation.
