# Supplementary material for: Immunomodulatory Macrophages Enable E-MNC Therapy for Radiation-Induced Salivary Gland Hypofunction
Source: Cells. 2023 May 17;12(10):1417. doi: 10.3390/cells12101417 (PMC10216929; doi:10.3390/cells12101417)
Supplement: Supplementary file 1 [file cells-12-01417-s001.zip › cells-2184912-supplementary.pdf]

Supplemental Table S1. Recombinant proteins of mouse 5G culture medium [8].

recombinant proteins	company, catalog No.	concentration
mouse SCF	Peprotech, 250-03	100 ng/ml
mouse Flt-3 ligand	Peprotech, 250-31L	100 ng/ml
mouse TPO	Peprotech, 315-14	20 ng/ml
mouse VEGF	Peprotech, 450-32	50 ng/ml
mouse IL-6	Peprotech, 216-16	20 ng/ml

Supplemental Table S2-1. Mouse antibodies for flow cytometric analysis.

antibodies	company, catalog No.
APC-Cy7 anti-mouse/human CD11b	Biolegend, 101225
APC/Cy7 Rat IgG2b, κ isotype Ctrl Antibody	Biolegend, 400623
APC anti-mouse CD206 (MMR)	Biolegend, 141707
APC Rat IgG2a, κ Isotype Ctrl Antibody	Biolegend, 400511
PE anti-mouse CD3	Biolegend, 100205
PE Rat IgG2b, κ isotype Ctrl Antibody	Biolegend, 400607
APC-Cy7 anti-mouse CD4	Biolegend, 100413
APC-Cy7 Rat IgG2b, κ isotype Ctrl Antibody	Biolegend, 400623
FITC anti-mouse/human CD45R/B220	Biolegend, 103205
FITC Rat IgG2a, κ isotype Ctrl Antibody	Biolegend, 400505
APC anti-mouse CCR2	R&D, FAB5538A
APC Rat IgG2b, κ isotype Ctrl Antibody	Biolegend, 400611
FITC anti-mouse Msr1(CD204)	BIO-RAD, MCA1322FT
FITC Rat IgG2b, κ isotype Ctrl Antibody	BD, 553988
FITC anti-mouse CD183 (CXCR3)	Biolegend, 126535
FITC Armenian hamster IgG, isotype Ctrl Antibody	Biolegend, 400905
PE/Cy7 anti-mouse CD194 (CXCR4)	Biolegend, 131213
PE/Cy7 Armenian hamster IgG, isotype Ctrl Antibody	Biolegend, 400921
APC anti-mouse CD196 (CCR6)	Biolegend, 129813
APC Armenian hamster IgG, isotype Ctrl Antibody	Biolegend, 400911
PE-Cy7 anti-mouse/human Mac-2 (Galectin-3)	Biolegend, 125417
PE-Cy7 Rat IgG2a, κ isotype Ctrl Antibody	Biolegend, 400521
True-Stain Monocyte Blocker™	Biolegend, 426102
PI	WAKO, 169-26281

Supplemental Table S2-2. Human antibodies for flow cytometric analysis.

antibodies	company, catalog No.
PE-Cy7 mouse IgG1, κ isotype Ctrl Antibody	Biolegend, 400126
PE-Cy7 anti-human CD11b	Biolegend, 301322
APC-Cy7 mouse IgG1, κ isotype Ctrl Antibody	Biolegend, 400128
APC-Cy7 anti-human CD206	Biolegend, 321120
FcR Blocking Reagent, human	Miltenyi Biotec, 130-059-901
PI	WAKO, 169-26281

Supplemental Table S3-1. Mouse primers.

gene	forward	reverse
IL-1β	5′-GCTGAAAGCTCTCCACCTCA-3′	5′-AGGCCACAGGTATTTTGTCG-3′
IL-10	5′-GCTGGACAACATACTGCTAACC-3′	5′-ATTTCCGATAAGGCTTGGCAA-3′
IFN-γ	5′-ACAGCAAGGCGAAAAAGGATG-3′	5′-TGGTGGACCACTCGGATGA-3′
VEGF-A	5′-CCTCCGAAACCATGAACTTT-3′	5′-TCATGGGACTTCTGCTCTCC-3′
IL-6	5′-CCACTCCCAACAGACCTGTC-3′	5′-GCAAGTGCATCATCGTTGTTC-3′
CD206	5′-CCACAGCATTGAGGAGTTTG-3′	5′-ACAGCTCATCATTTGGCTCA-3′
IGF1	5′-CACACCTCTTCTACCTGGCG-3′	5′-AGCCTGTGGGCTTGTTGAAG-3′
TGF-β	5′-GGACTCTCCACCTGCAAGAC-3′	5′-GACTGGCGAGCCTTAGTTTG-3′
TLR2	5′-AACTCTGACCCGCCCTTTAA-3′	5′-AGCCTGAAGTGGGAGAAGTC-3′
TLR4	5′-CTGCATAGAGGTAGTTCCTA-3′	5′-AATTTCACACCTGGATAAAT-3′
NGF	5′-ATGGGGGAGTTCTCAGTGTG-3′	5′-GCACCCACTCTCAACAGGAT-3′
Car3	5′-CTTGATGCCCTGGACAAAAT-3′	5′-AGCTCACAGTCATGGGCTCT-3′
MMP9	5′-CGTCATTCGCGTGGATAAGG-3′	5′-TTTGGAAACTCACACGCCAG-3′
GAPDH	5′-TGCACCACCAACTGCTTAG-3′	5′-GGATGCAGGGATGATGTTC-3′

Supplemental Table S3-2. Human primers.

gene	forward	reverse
IL-1β	5′-CACAGACCTTCCAGGAGAAT-3′	5′-TTCAACACGCAGGACAGGTA-3′
TNF-α	5′-AATGGCGTGGAGCTGAGA-3′	5′-TAGACCTGCCCAGACTCGG-3′
IFN-γ	5′-CTGTTACTGCCAGGACCCAT-3′	5′-ACACTCTTTTGGATGCTCTGGT-3′
GAPDH	5′-GGAGTCCACTGGCGTCTTCAC-3′	5′-GCTGATGATCTTGAGGCTGTTGTC-3′

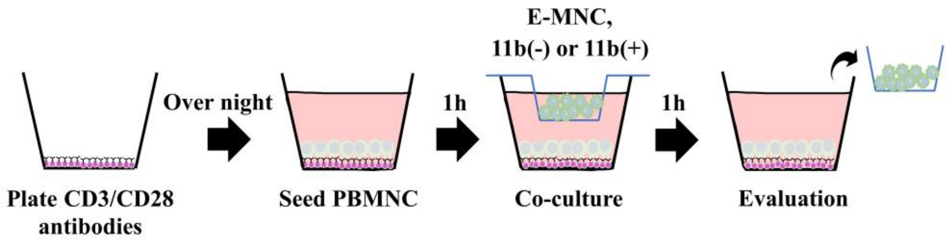

Supplemental Figure S1. Schematic diagram describing the experimental design for co-culture of E-MNCs and T-cell–activated PBMNCs. Anti-CD3 and -CD28 antibodies were added to the wells of a 24-well plate and incubated overnight. Then, PBMNCs were seeded to the wells and cultured at 37°C for 1 h. Subsequently, E-MNCs, 11b(−) cells, or 11b(+) cells were seeded to the upper chamber and co-cultured with stimulated PBMNCs at 37°C for 1 h. Finally, co-cultured PBMNCs were collected and analyzed.

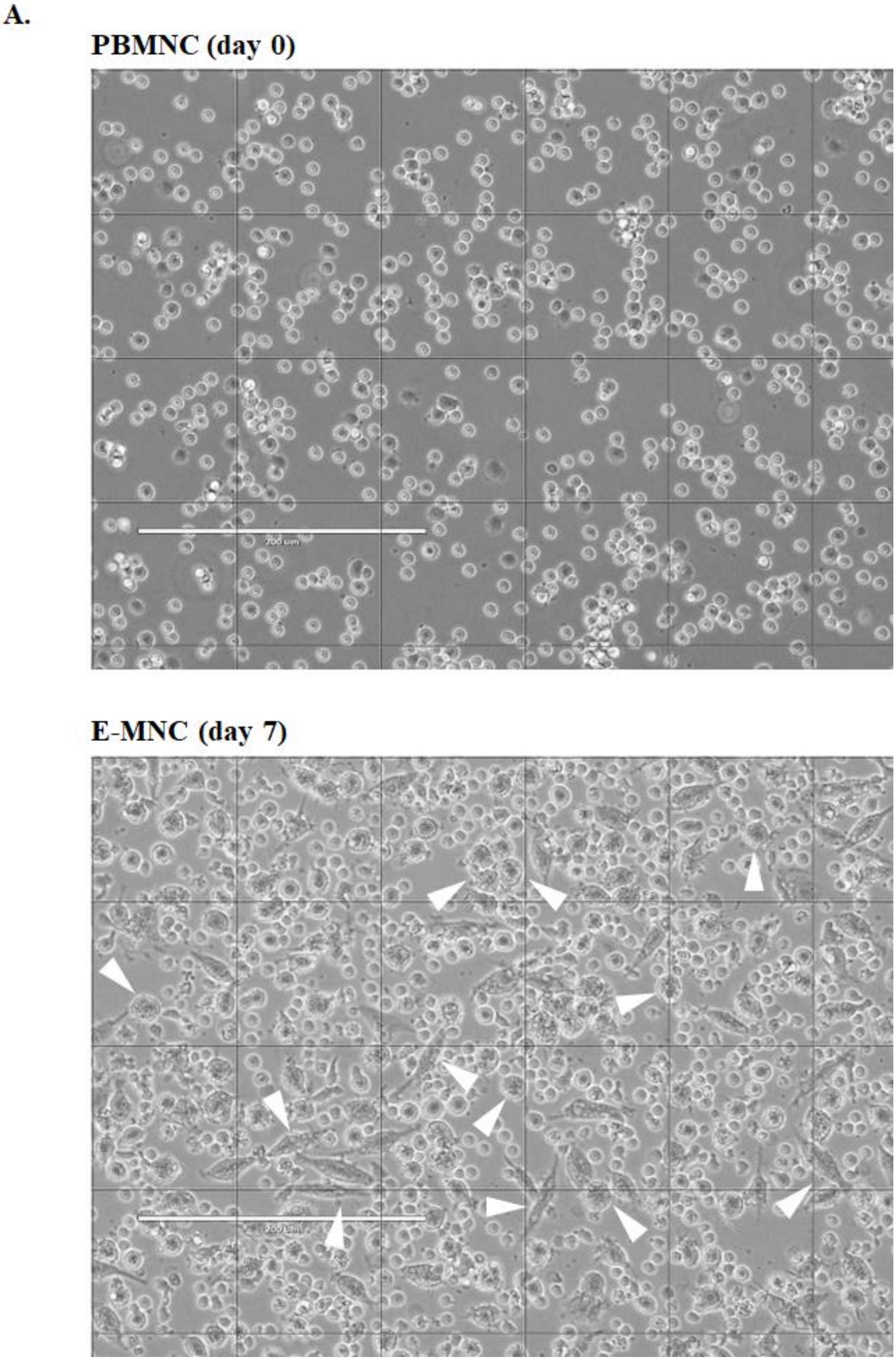

Supplemental Figure S2. Characteristics of human E-MNCs. (A) Phase-contrast imaging of PBMNCs (day 0) and E-MNCs (day 7). White arrows indicate round- and spindle-shaped macrophage-like cells (white arrows). Scale bar; 200 μ m.

A.

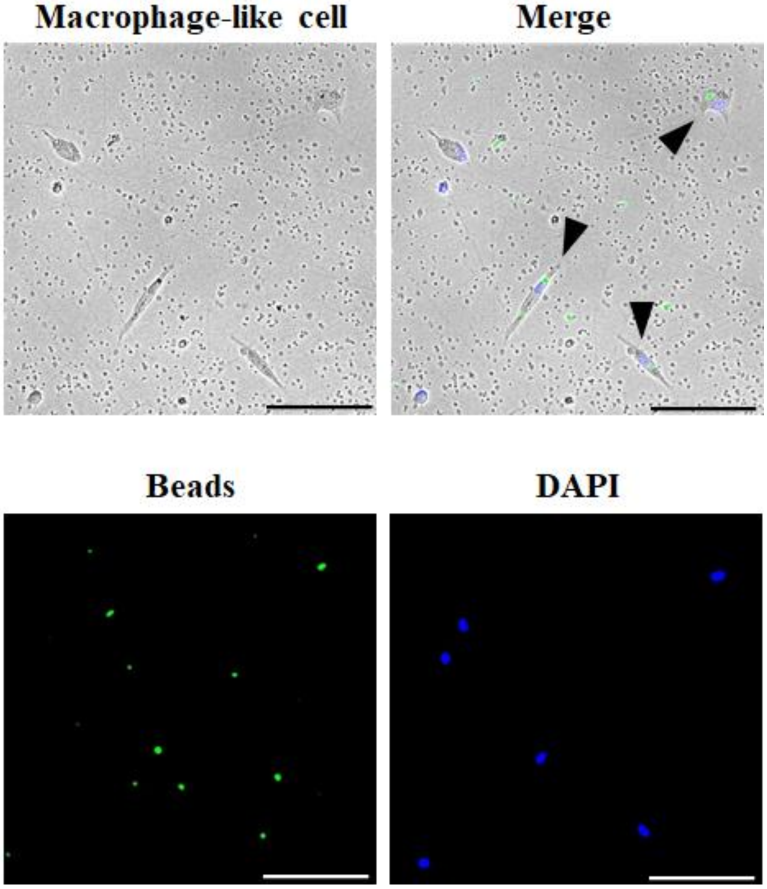

Supplemental Figure S3. Characteristics of human E-MNCs. **(A)** Phagocytosis assay with fluorescent beads. Phase-contrast and fluorescence microscopic images of macrophage-like round- and spindle-shaped cells (black arrows) among E-MNCs at 1 h. Green, fluorescent beads; Blue, DAPI. Scale bar 100µm.

A.

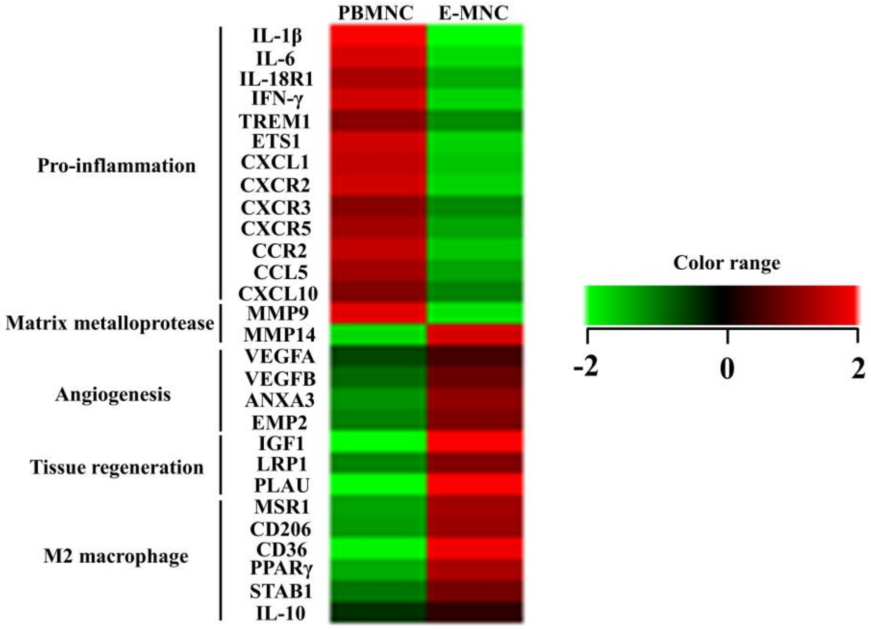

Supplemental Figure S4. Microarray analysis of PBMNCs and E-MNCs. **(A)** Heat map showing down-regulated and up-regulated genes associated with inflammation, matrix metalloproteases, angiogenesis, tissue regeneration, and M2-macrophages in PBMNCs (day 0) and E-MNCs (day 5). Red color indicates relatively higher expression, and green color indicates relatively lower expression.

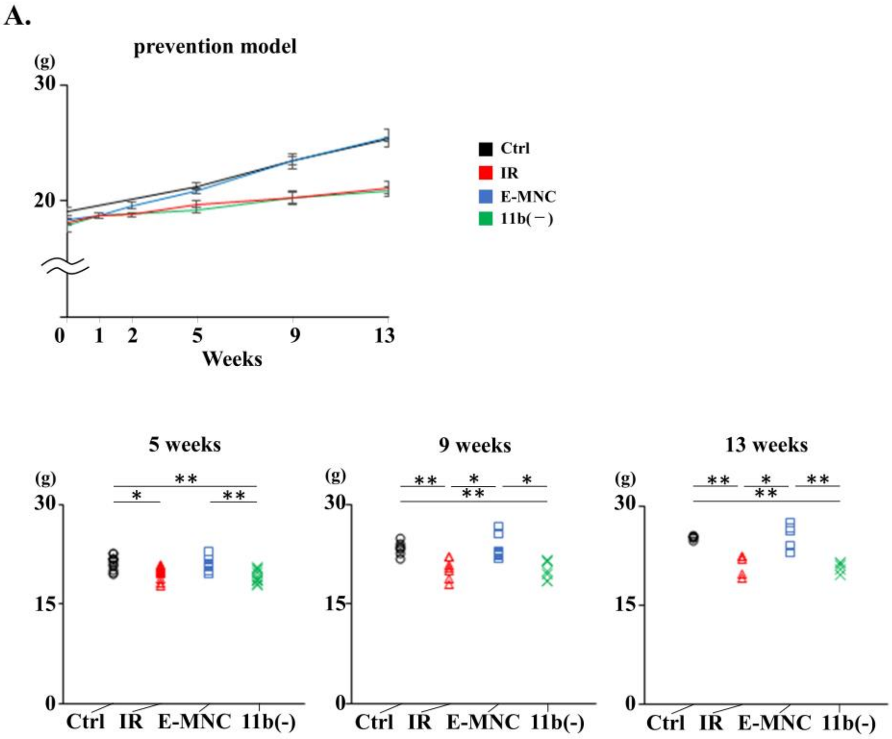

Supplemental Figure S5. Changes in body weight in the prevention mouse model after transplantation. **(A)** Change in body weight in each group at 1, 2, 5, 9, and 13 weeks post-IR (upper graph) and scatter diagram of body weight in each specimen at 5, 9, and 13 weeks post-IR (lower graphs) (*P<0.05, **P<0.01).

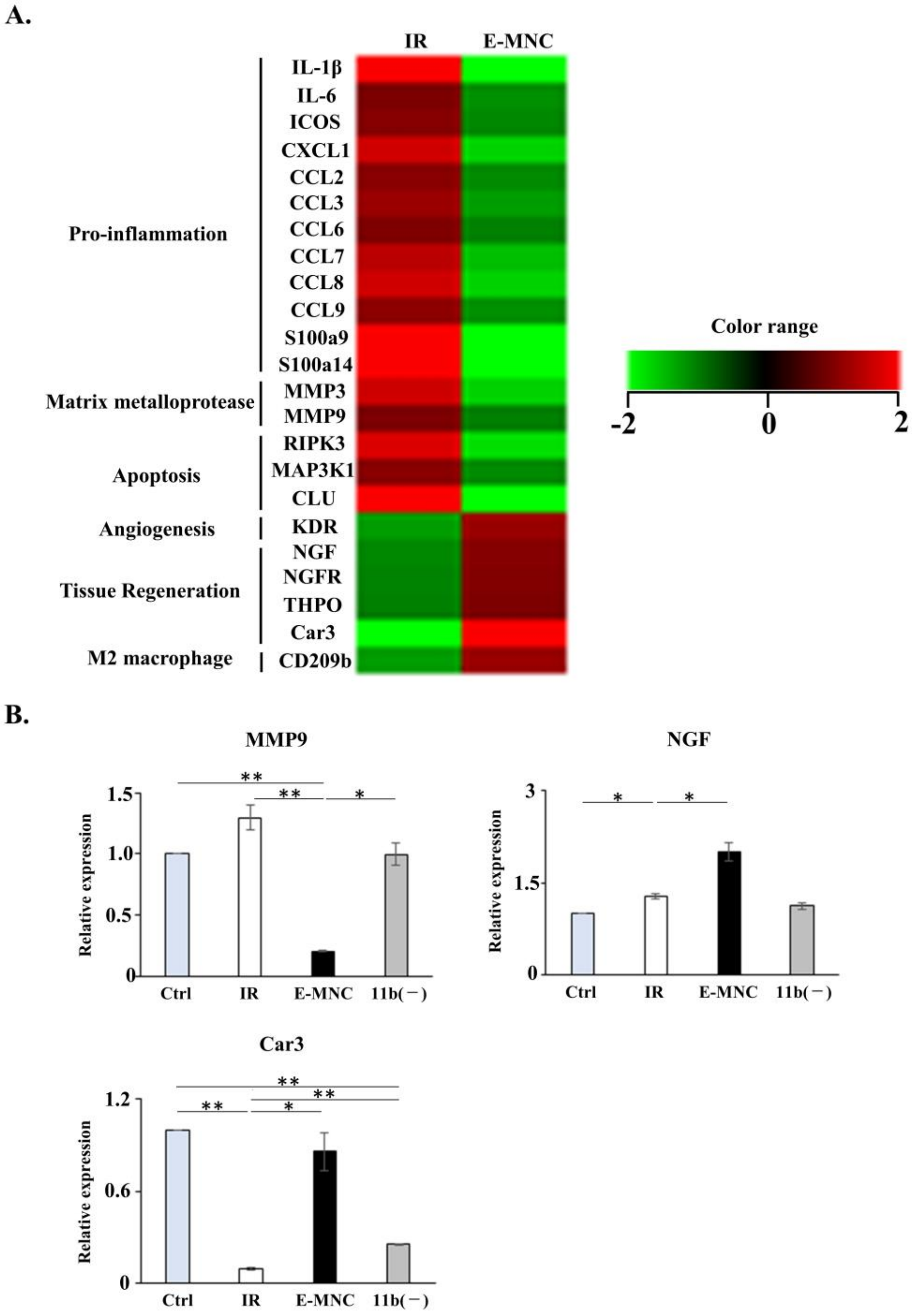

Supplemental Figure S6. Microarray and qPCR analyses of transplanted specimens at 2 weeks post-IR. **(A)** Heat map showing down-regulated and up-regulated genes associated with inflammation, matrix metalloproteases, apoptosis, angiogenesis, tissue regeneration, and M2-macrophages in the IR- and E-MNC-groups. Red color indicates relatively higher expression, and green color indicates relatively lower expression. **(B)** Expression of mRNAs of genes encoding MMP9, NGF, and Car3 in each group (*P<0.05, **P<0.01).

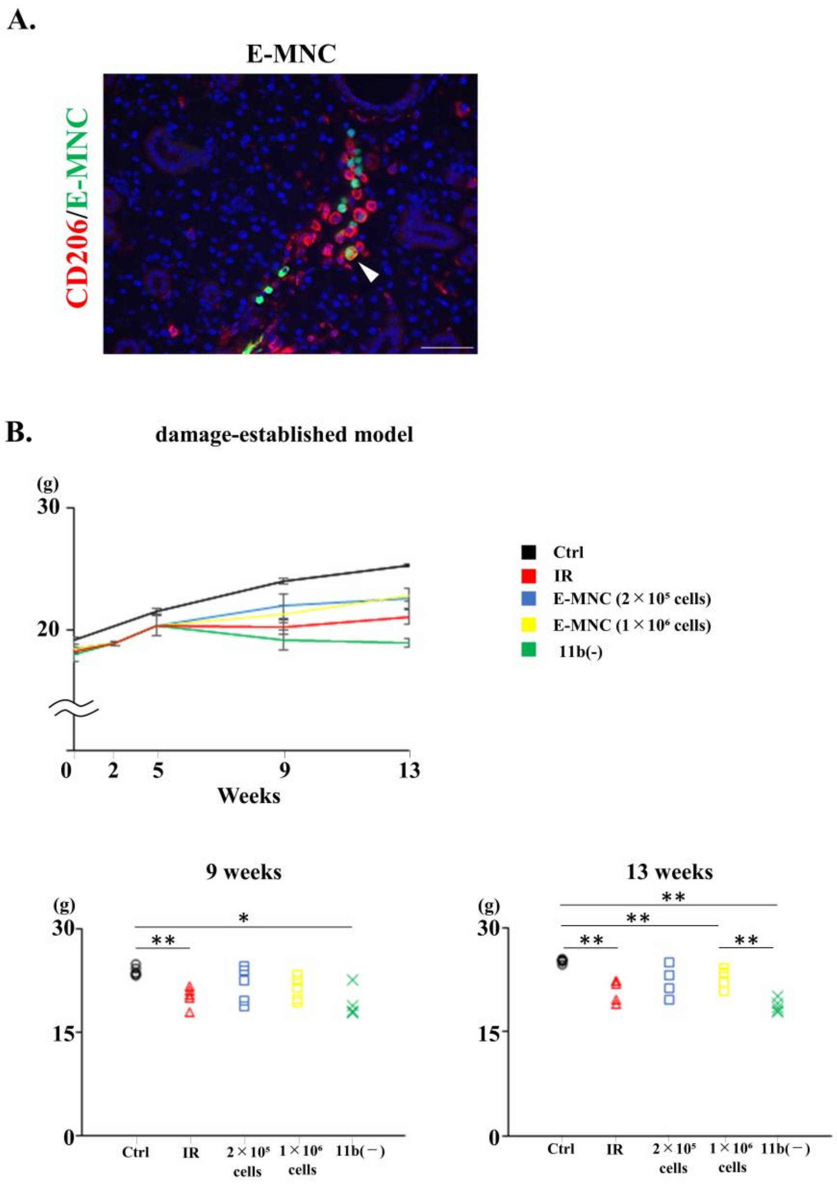

Supplemental Figure S7. Immunohistological observations at 10 days post-IR and changes in body weight after transplantation. **(A)** CD206-expressing cells (red) at the periphery of transplanted E-MNCs (green). Blue, DAPI. Scale bar, 50 µm. (B) Changes in body weight in each group of the established model at 2, 5, 9, and 13 weeks post-IR (upper graph), and scatter diagram of body weight in each specimen at 9 and 13 weeks post-IR (lower graphs) (**P*<0.05, ***P*<0.01).
